# 

**Published:** 1911-01-14

**Authors:** 


### THE HOSPITAL

Telegraphic Address: Ospedale, London.

THE MODERN MEDICAL AND INSTITUTIONAL JOURNAL.

Telephone Number: 2734 Gerrard.

NEW SERIES. No. 206, Vol. VIII.

No. 1278, Vol. XLIX.

SATURDAY, JAN. 14TH, 1911.

PRICE THREEPENCE

